# Supplementary material for: Compositionally-distinct ultra-low velocity zones on Earth’s core-mantle boundary
Source: Nat Commun. 2017 Aug 2;8:177. doi: 10.1038/s41467-017-00219-x (PMC5540928; doi:10.1038/s41467-017-00219-x)
Supplement: Supplementary file 4 — Supplementary Information [file 41467_2017_219_MOESM4_ESM.pdf]

File name: Supplementary Information
Description: Supplementary Figures, Supplementary Tables, Supplementary Notes and Supplementary References

File name: Supplementary Movie 1
Description: Time evolution of distribution of hottest regions (orange) respected to the thermochemical piles (green) throughout the model run from 0 to 506 Myrs. The model boundaries are shown by dark gray color and the CMB is shown by light gray color.

File name: Supplementary Movie 2
Description: Time evolution of distribution of ultra-dense material (orange) respected to the thermochemical piles (green) throughout the model run from 0 to 436 Myrs. The model boundaries are shown by dark gray color and the CMB is shown by light gray color.

File name: Peer Review File
Description:

Supplementary Note 1 | Cooling the pile edges by thermal diffusion

Here, we performed 2 two-dimensional isochemical calculations (Case S1 and Case S2) to study the process of cooling the edges of piles by thermal diffusion. In these 2D models, temperature is isothermal both on the top (T=0.0) and at the bottom (T=1.0). All boundaries are free slip. Viscosity is temperature dependent with $\eta = \exp[\mathrm{A}(0.5 - T)]$, where the activation coefficient A=6.91, leading to 1,000 viscosity change due to changes of temperature. In addition, a viscosity increases of 50x is employed from upper mantle to lower mantle. Both cases have a Rayleigh number of Ra=1e6. The model has an aspect ratio of 1 and is divided into 128x128 elements.

All physical parameters of both cases are the same, except that for Case S1, both side boundaries are insulating, while for Case S2, the right boundary is insulating but the left boundary is isothermal with a temperature of T=0.5, which serves as a 'cooling' boundary. Supplementary Fig. 2 shows snapshots of temperature field for these two cases when it reaches steady state. For Case S1 (Supplementary Fig. 2a), single cell convection is established with cold downwellings occur at the right boundary and hot upwellings at the left boundary. Hot thermal instabilities in the lowermost mantle are advected to the left side boundary. In this case, the hottest regions occur at the lower left of the model. However, when a cooling left boundary is employed (Case S2, Supplementary Fig. 2b), the hottest regions are located away from the left boundary, because of cooling by the left boundary.

Similarly, the edges of piles are cooled down by thermal diffusion from surrounding cold background mantle, and as a result, the hottest region appears inboard from the edges of piles.

Supplementary Note 2 | Other geodynamic models

In Case 2, we initially introduced a global layer of ultra-dense material above the CMB with a thickness of 5 km and with a buoyancy number of B=2.0. One may argue that the size of the accumulations of ultra-dense material depends on the initial volume of ultra-dense material that we introduce to the model, and their shape may be affected by the intrinsic density of the ultra-dense material. To explore how the initial volume and intrinsic density of the ultra-dense material affect the morphology and distribution of the ultra-dense material, we perform two more cases, Case 3 and Case 4. In Case 3, the intrinsic density of the ultra-dense material is increased and the ultra-dense material has a buoyancy number of B=3.0, or is ~13.5% intrinsically denser than the background mantle material. In Case 4, the initial thickness of the global layer of ultra-dense material is increased to 10 km, which is twice that of Case 2. As expected, by increasing the intrinsic density (Case 3) or the initial volume of the ultra-dense material (Case 4), the size of the accumulations of ultra-dense material increases (Supplementary Fig. 3-4). However, like Case 2, most ultra-dense materials accumulates into discontinuous patches along the edges of thermochemical piles in Cases 3 and 4, with variable size and shape.

Several previous studies have suggested that the ULVZs may be enriched in iron[1-3]. The Fe-enriched ULVZs may have low solidus temperature[4-6] and may be partially molten. If so, the viscosity of the partially molten ULVZs is expected to be reduced. However, we don't envision a typical scenario in which ULVZs would have a higher intrinsic viscosity than the surroundings. It cannot be ruled out that ULVZs could have significantly larger grain-size (relative to surroundings) which would act to increase their diffusion creep viscosity. However, the associated viscosity increase would have to overcome the strong viscosity decrease due to temperature-dependence. In any case, we consider this possibility to be beyond the scope of the present manuscript. In Case 5,

the viscosity of ultra-dense material is further reduced by 100x in addition to temperature dependent viscosity. We find more stirring between the ultra-dense material and pile material in this case (Supplementary Fig. 5) than Case 2. Nonetheless, the distribution of ultra-dense materials in Case 5 is similar to that in Case 2, and they form into discontinuous patches along the edges of thermochemical piles, and most ultra-dense materials occur near the pile edges (Supplementary Fig. 5).

In Case 6, we use a larger activation coefficient (A=11.51) than in Case 2, which leads to 5 orders of maximum viscosity contrast due to temperature. As a result, the viscosity contrast between thermochemical piles (including the ultra-dense materials) and their surrounding mantle is increased, which results in a reduction of viscous coupling between the piles (including the ultra-dense materials) and surrounding mantle[7]. This may be the reason why the accumulation of ultra-dense materials in Case 6 is, in general, slightly larger than that in Case 2 (Supplementary Fig. 6). Nevertheless, the distribution of ultra-dense materials in Case 6 is like that in Case 2, with most ultra-dense materials occurring at the edges of thermochemical piles (Supplementary Fig. 6).

The preferential occurrence of ultra-dense material near the edges of thermochemical piles in Case 2-6 is not a coincidence of the large-scale mantle flow pattern in these cases. In Case 7, we use a higher Rayleigh number but a lower intrinsic density for the pile material. As expected, the convection becomes more vigorous under higher Rayleigh number and the wavelength for the convective structure is shorter than in Case 2 (Supplementary Fig. 7). Although the morphology of the thermochemical piles in Case 7 is different from that in Case 2, most ultra-dense materials accumulate into discontinuous patches at the edges of thermochemical piles in Case 7, similar to the results for Case 2. In Case 8, we increase the intrinsic density of pile material. As expected, the thermochemical piles cover slightly more CMB area than in Case 2. However, the distribution

of ultra-dense material is similar to that in Case 2, with the majority of ultra-dense materials occurring near the edges of thermochemical piles and forming into discontinuous patches (Supplementary Fig. 8).

We also explored how changes of Rayleigh number, temperature dependent viscosity and properties of pile material affect the locations of hottest regions (Cases 9-11). Although the morphology of thermochemical piles may differ case by case (which has also been discussed in details in ref. 7 and ref. 8), the hottest lowermost mantle regions are generally located well within the interiors of thermochemical piles for all of these cases (Supplementary Fig. 9-10). It is worth mentioning that the maximum distance of hottest regions from the edges of thermochemical piles is controlled by the size of the piles. For example, we use a higher Rayleigh number but a lower intrinsic density for the pile material in Case 10. Similar to Case 7, the convective length is reduced with increased Rayleigh number. In contrast to the large thermochemical piles in the lowermost mantle in cases with smaller Rayleigh number (e.g., Case 1), there are several cold downwelling regions in the lowermost mantle that separate the pile materials into relatively small interconnect piles for Case 10 (Supplementary Fig. 10 a, b). As a result, the maximum distance between edges of the thermochemical piles and the hottest regions is smaller for Case 10 than other cases with smaller Rayleigh number. The distances of hottest regions from the pile edges for Case 10 are also generally smaller than other cases with smaller Rayleigh number, but the hottest regions in Case 10 are still well within the thermochemical piles (Supplementary Fig. 10 a, b).

Supplementary Table 1 | All cases used in this study

Case	B_u	D_u	μ_u	A	Ra	B_p	Fig.
1	N/A	N/A	N/A	9.21	9.8e6	0.8	1
2	2.0	5 km	1.0	9.21	9.8e6	0.8	3, 4
3	**3.0**	5 km	1.0	9.21	9.8e6	0.8	S3
4	2.0	**10 km**	1.0	9.21	9.8e6	0.8	S4
5	2.0	5 km	**0.01**	9.21	9.8e6	0.8	S5
6	2.0	5 km	1.0	**11.51**	9.8e6	0.8	S6
7	2.0	5 km	1.0	9.21	**4.8e7**	**0.6**	S7
8	2.0	5 km	1.0	9.21	9.8e6	**1.0**	S8
9	N/A	N/A	N/A	**11.51**	9.8e6	0.8	S9
10	N/A	N/A	N/A	9.21	**4.8e7**	**0.6**	S10a,b
11	N/A	N/A	N/A	9.21	9.8e6	**1.0**	S10c,d

B_u : Buoyancy number of ultra-dense material; D_u: Initial thickness of the global layer of ultra-dense material; μ_u: Composition dependence of viscosity reduction of ultra-dense material; A: Activation coefficient; Ra: Rayleigh number. Parameters of cases with three compositional components are shown in green color. Parameters of cases with two compositional components are shown in red color. Numbers in bold are parameters whose value is different from Case 1 or Case 2.

Supplementary Table 2 | Parameters used in this study

Parameters	Reference value
Mantle thickness	2890 km
Mantle density ρ_0	5500 kg/m^3
Thermal expansivity α_0	1.8×10^{-5} K^{-1}
Thermal diffusivity κ_0	1×10^{-6} m^2/s
Gravitational acceleration g	9.8 m/s^2
Temperature change across the mantle ΔT	2500 K
Reference viscosity η_0	6×10^{21} Pa s

Supplementary Table 3 | Reflected wave ULVZ studies used

Ref.	Study	Seismic wave used	Region
9	Zhao et al. [2017]	ScS	Central America
10	Gassner et al. [2015]	PcP	Europe
11	Idehara [2011]	ScP	Philippines
12	Rost et al. [2010]	ScP	Coral Sea
13	Hutko et al. [2009]	PcP	Central America
14	He & Wen [2009]	ScS	West Pacific
15	Idehara et al. [2007]	ScP	Celebes Sea; Philippine Sea; Coral Sea.
16	Rost et al. [2006]	ScP	Coral Sea
17	Avants et al. [2006]	ScS	Central Pacific
18	Rost et al. [2005]	ScP	Coral Sea
19	Ross [2004]	PcP	North & West Siberia
20	Koper [2004]	PcP	Coral Sea
21	Rost [2003]	ScP	Coral Sea
22	Rost [2001]	ScP	Coral Sea
23	Ni & Helmberger [2001]	ScS	South Atlantic
24	Havens & Revenaugh [2001]	PcP	Central America
25	Reasoner & Revenaugh [2001]	ScP	Southwest Pacific
26	Garnero & Vidale [1999]	ScP	Southwest Pacific
27	Kohler et al. [1997]	PcP	Central Pacific
28	Revenaugh [1997]	PcP	Alaska; Central Pacific
29	Mori & Helmberger [1995]	PcP	Central Pacific

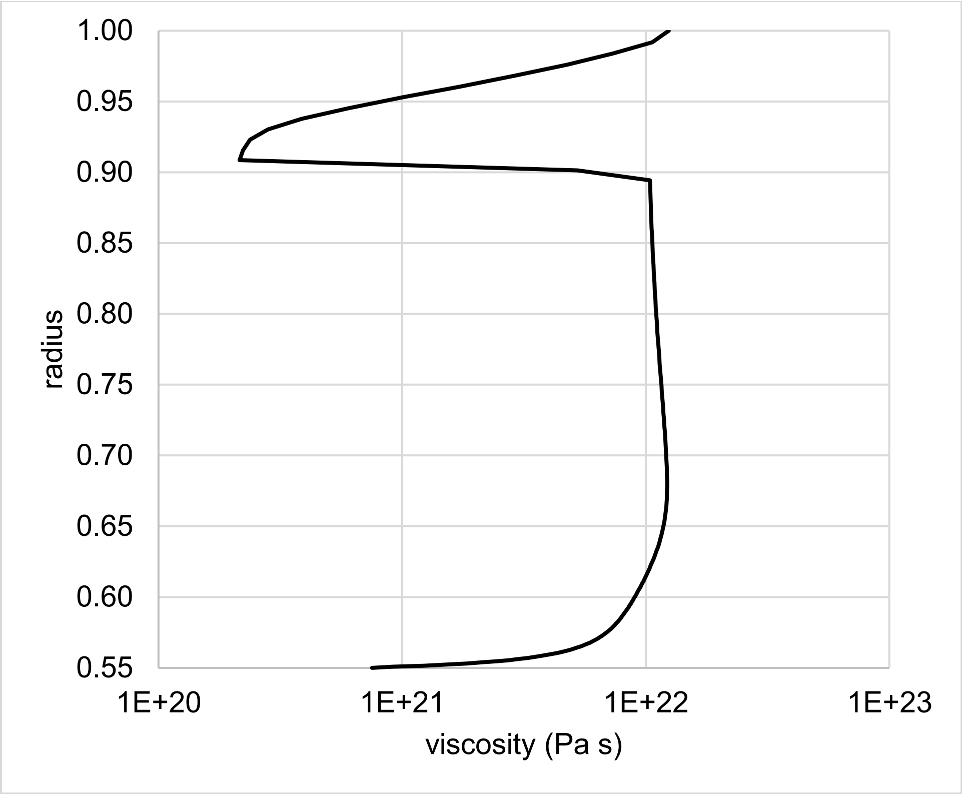

Supplementary Figure 1 | The horizontally averaged radial viscosity profile at initial condition.

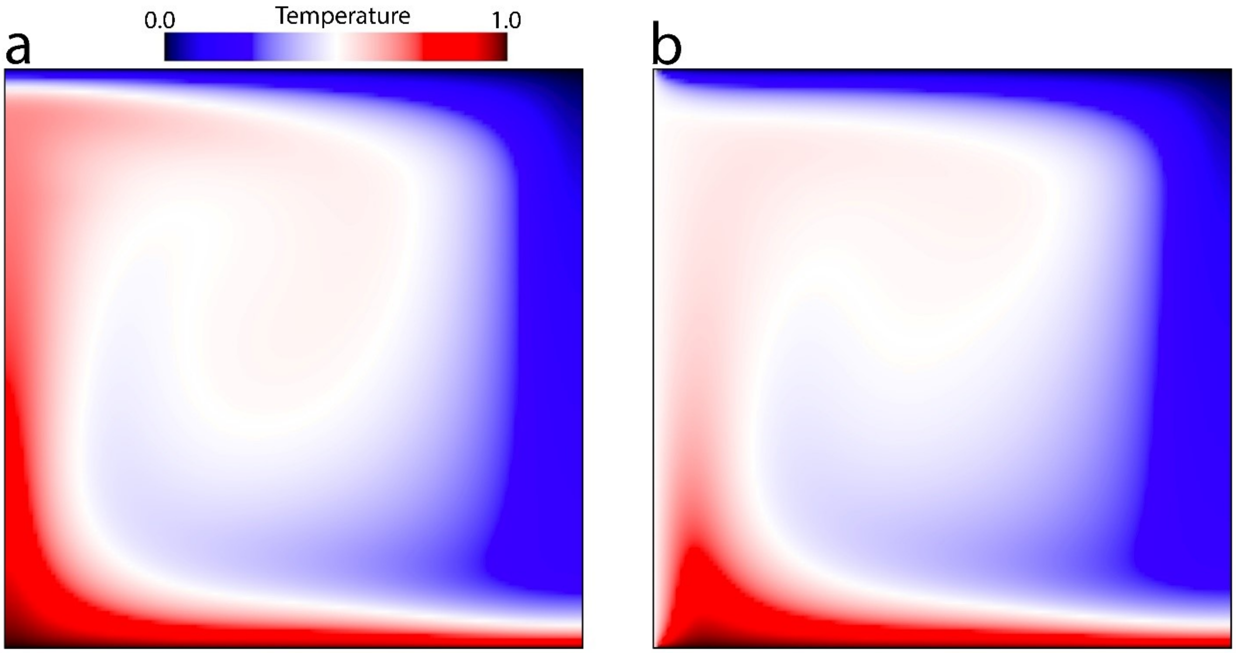

Supplementary Figure 2 | Cooling the pile edges by thermal diffusion. Steady state temperature field for (**a**) Case S1 and (**b**) Case S2. For Case S1, both side boundaries are insulating. For Case S2, the right boundary is insulating while the left boundary is isothermal with a temperature of T=0.5. Notice that for Case S1, the hottest regions occur at the lower left corner of the model. For Case S2, the hottest regions are located off the left boundary.

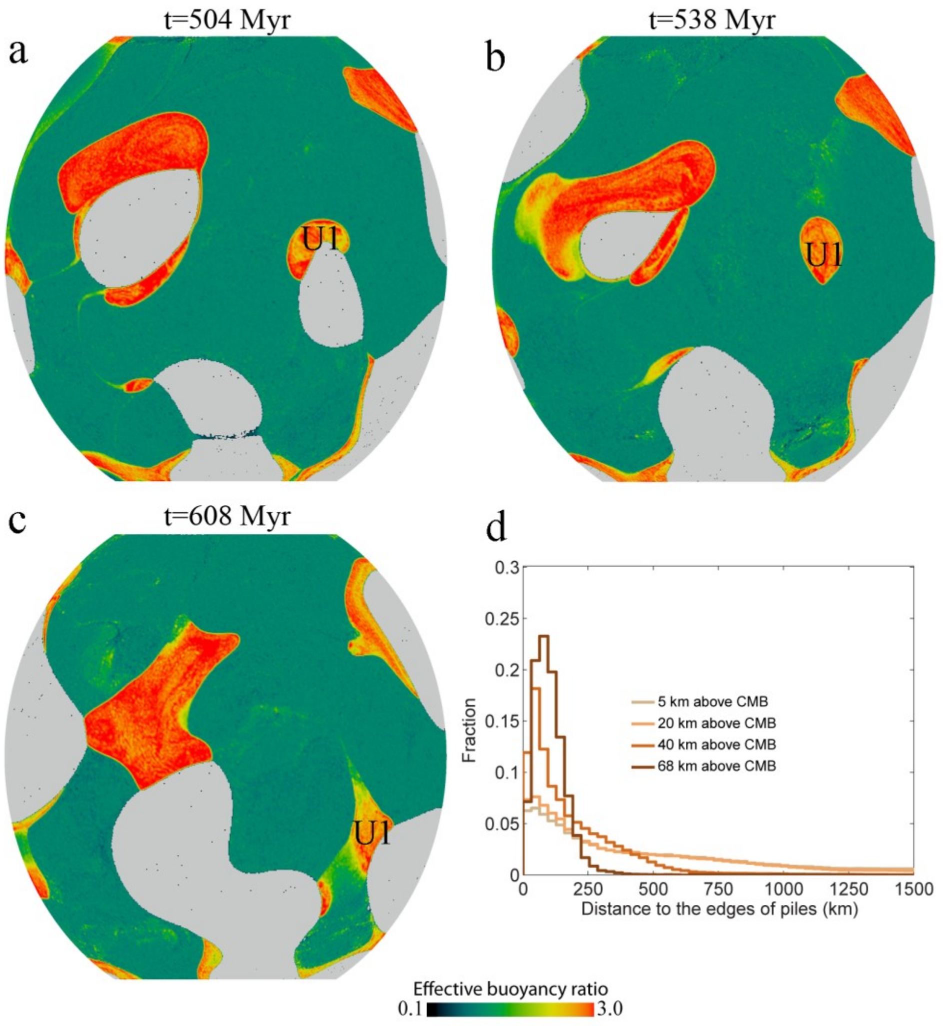

Supplementary Figure 3 | Effects of intrinsic density for ultra-dense material on the morphology and distribution of ultra-dense material. a-c, snapshots showing the composition field at 5 km above the CMB superimposed with mantle flow velocity arrows for Case 3, in which the buoyancy number of ultra-dense material is B=3.0. Red: ultra-dense material; green to yellow: stirring between ultra-dense material and pile material. Gray color shows the exposed CMB. **a**, accumulation of ultra-dense material (U1) is located at the edges of piles. **b**, U1 is located in the center of piles after the branches of piles merge together. **c**, U1 is moved to the edges of piles. **d**, Fraction of ultra-dense material for variable distances from the closest edges of piles.

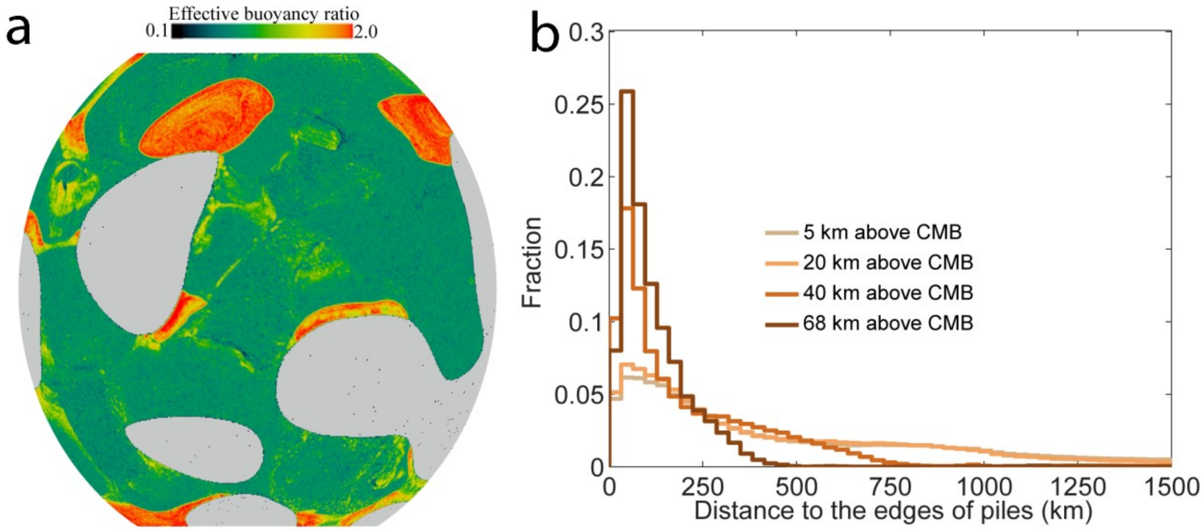

Supplementary Figure 4 | Effects of volume of ultra-dense material on the morphology and distribution of ultra-dense material. a, snapshot of compositional field at 5 km above the CMB for Case 4 at 462 Myr, in which the initial thickness of the global layer of ultra-dense material is 10 km. Red: ultra-dense material; green to yellow: stirring between ultra-dense material and pile material. Gray color shows the exposed CMB. **b**, Fraction of ultra-dense material for variable distances from the closest edges of piles.

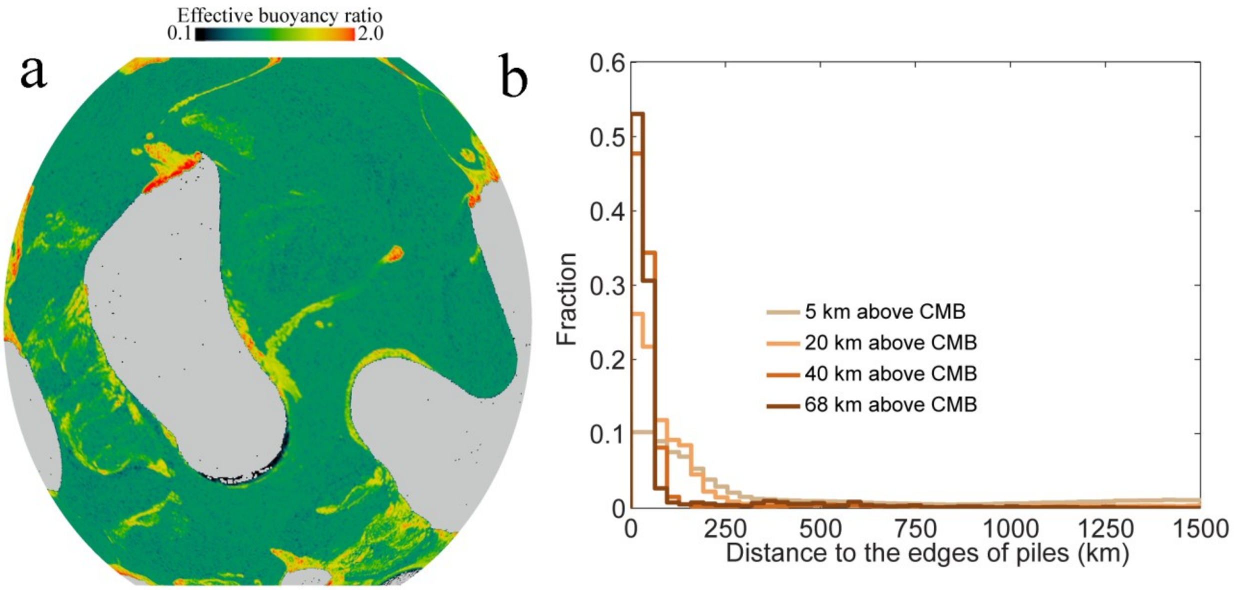

Supplementary Figure 5 | Effects of intrinsic compositional viscosity for ultra-dense material on the morphology and distribution of ultra-dense material. **a**, snapshot of compositional field at 5 km above the CMB for Case 5 at 144 Myr, in which a 100x viscosity reduction (in addition to the reduction due to temperature-dependence) is applied to the ultra-dense material, in addition to temperature dependent viscosity. Red: ultra-dense material; green to yellow: stirring between ultra-dense material and pile material. Gray color shows the exposed CMB. **b**, Fraction of ultra-dense material for variable distances from the closest edges of piles.

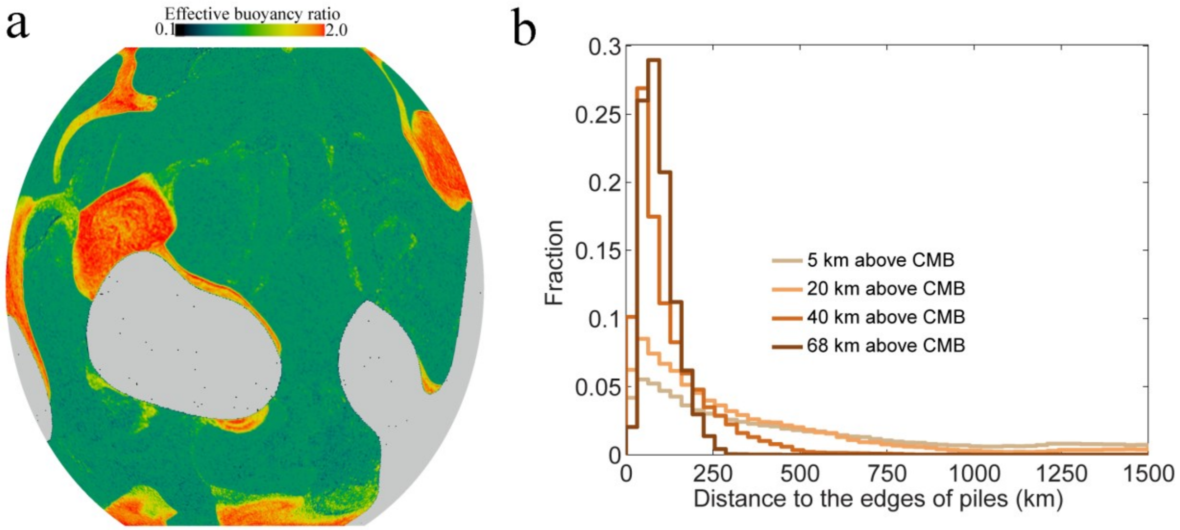

Supplementary Figure 6 | Effects of temperature dependent viscosity on the morphology and distribution of ultra-dense material. a, snapshot of compositional field at 5 km above the CMB for Case 6 at 166 Myr, in which the temperature dependent of viscosity is *A*=11.51, leading to 100,000x viscosity contrast due to temperature. Red: ultra-dense material; green to yellow: stirring between ultra-dense material and pile material. Gray color shows the exposed CMB. **b**, Fraction of ultra-dense material for variable distances from the closest edges of piles.

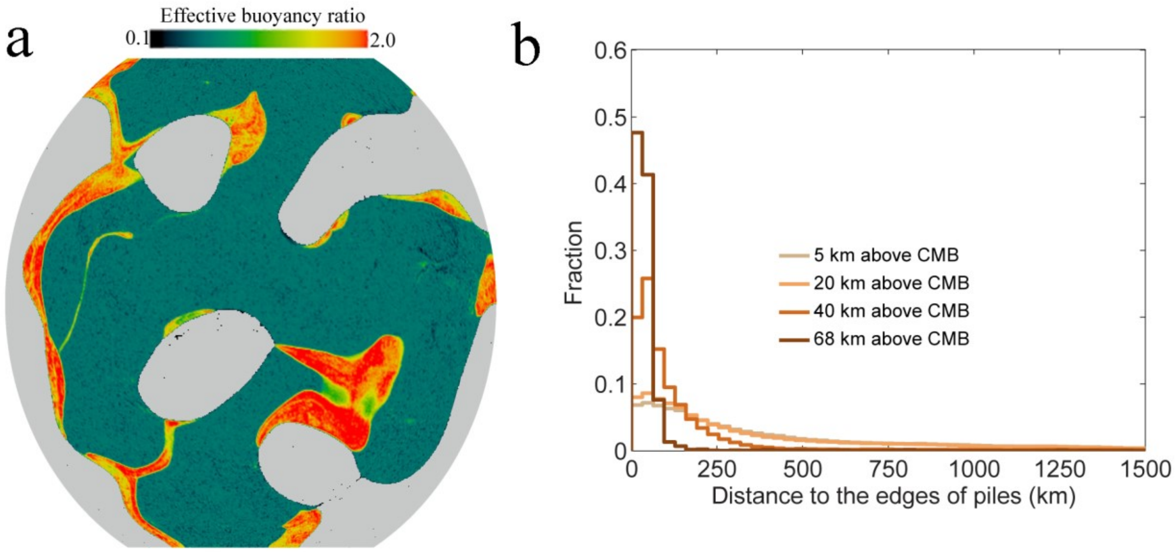

Supplementary Figure 7 | Effects of Rayleigh number on the morphology and distribution of ultra-dense material. a, snapshot of compositional field at 5 km above the CMB for Case 7 at 466 Myr, in which the Rayleigh number is Ra=4.8e7 and the buoyancy number for the pile material is $B_{\mathrm{p}} = 0.6$. Red: ultra-dense material; green to yellow: stirring between ultra-dense material and pile material. Gray color shows the exposed CMB. **b**, Fraction of ultra-dense material for variable distances from the closest edges of piles.

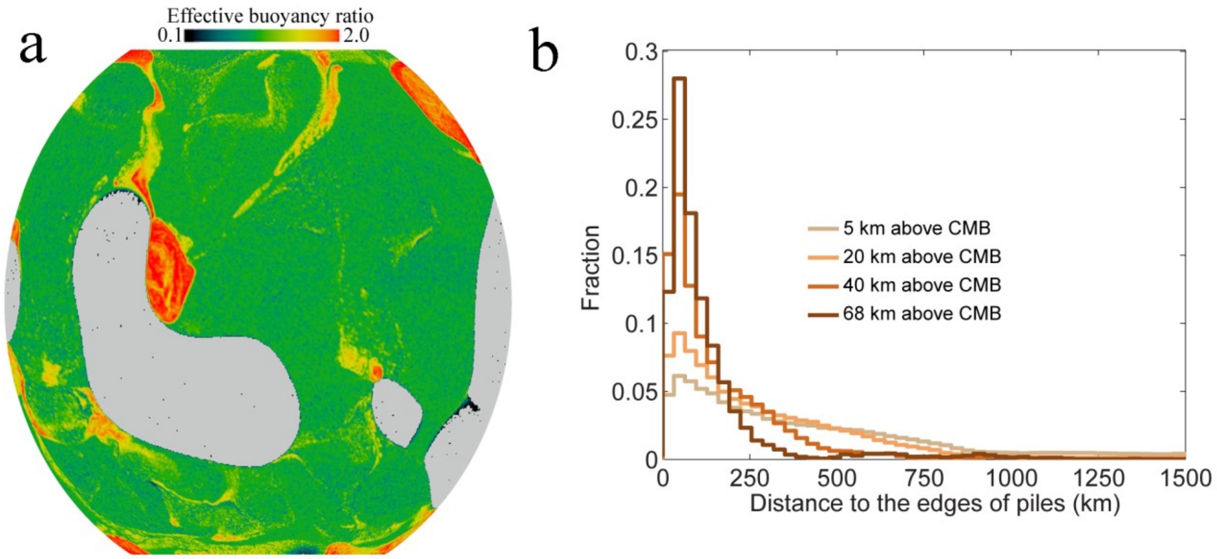

Supplementary Figure 8 | Effects of intrinsic density for thermochemical piles on the morphology and distribution of ultra-dense material. a, Snapshot of compositional field at 5 km above the CMB for Case 8 at 281 Myr, in which the buoyancy number for the pile material is $B_{\mathrm{p}} = 1.0$. Red: ultra-dense material; green to yellow: stirring between ultra-dense material and pile material. Gray color shows the exposed CMB. **b**, Fraction of ultra-dense material for variable distances from the closest edges of piles.

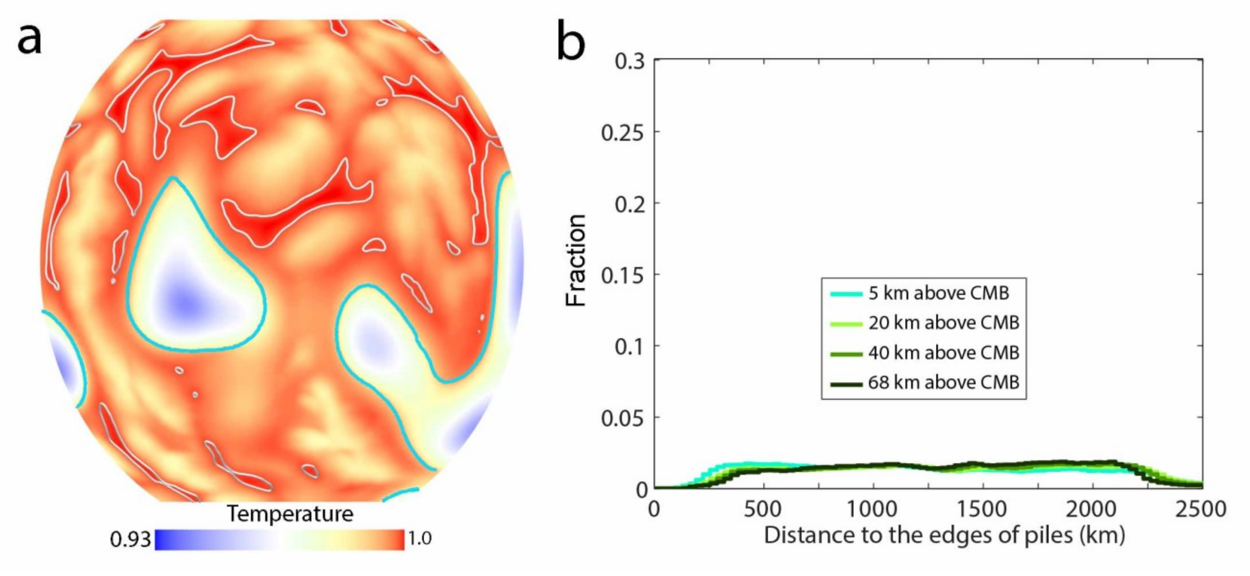

Supplementary Figure 9 | Effects of temperature dependent viscosity on the morphology and distribution of hottest regions. a, Snapshot of temperature field at 5 km above the CMB for Case 9 at 108 Myr, in which the temperature dependent of viscosity is A=11.51, leading to 100,000x visocisty contrast due to temperature. The hottest 10% regions of the piles by area are shown by light gray contours and the edges of piles are shown by cyan lines. **b**, Fraction of hottest regions by area for variable distances from the closest edges of piles.

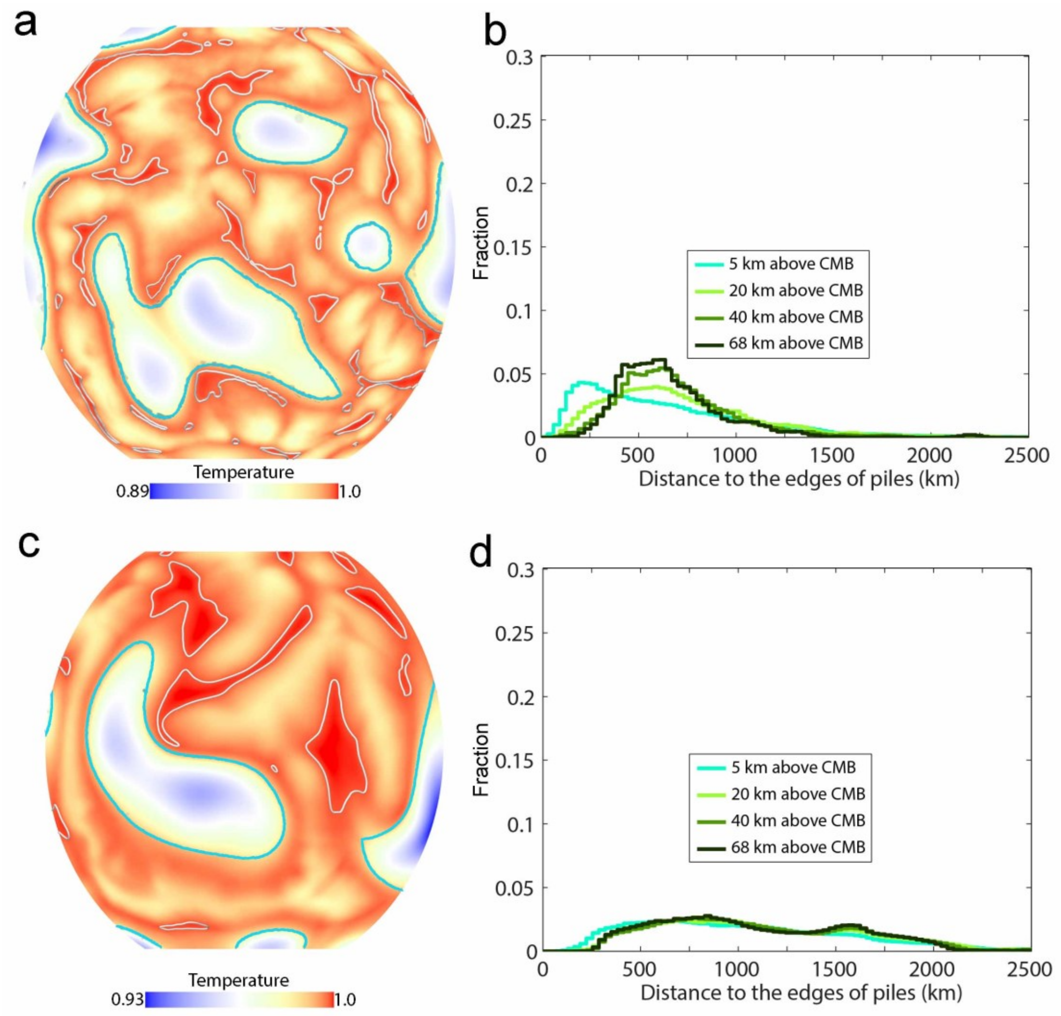

Supplementary Figure 10 | Effects of Rayleigh number and intrinsic density of thermochemical piles on the morphology and distribution of hottest regions. a, Temperature field at 5 km above the CMB for Case 10 at 393 Myr, in which the Rayleigh number is Ra=4.8e7 and the buoyancy number for the thermochemical pile (LLVP) material is $B_{\mathrm{p}} = 0.6$. **b**, Fraction of hottest regions by area for variable distances from the closest pile edges for Case 10. **c**, Temperature field at 5 km above the CMB for Case 11 at 312 Myr, in which the buoyancy number for the pile material is $B_{\mathrm{p}} = 1.0$. **d**, Fraction of hottest regions by area for variable distances from the closest pile edges for Case 11. For **a** and **c**, the hottest 10% regions of piles by area are shown by light gray contours and the edges of piles are shown by cyan lines.

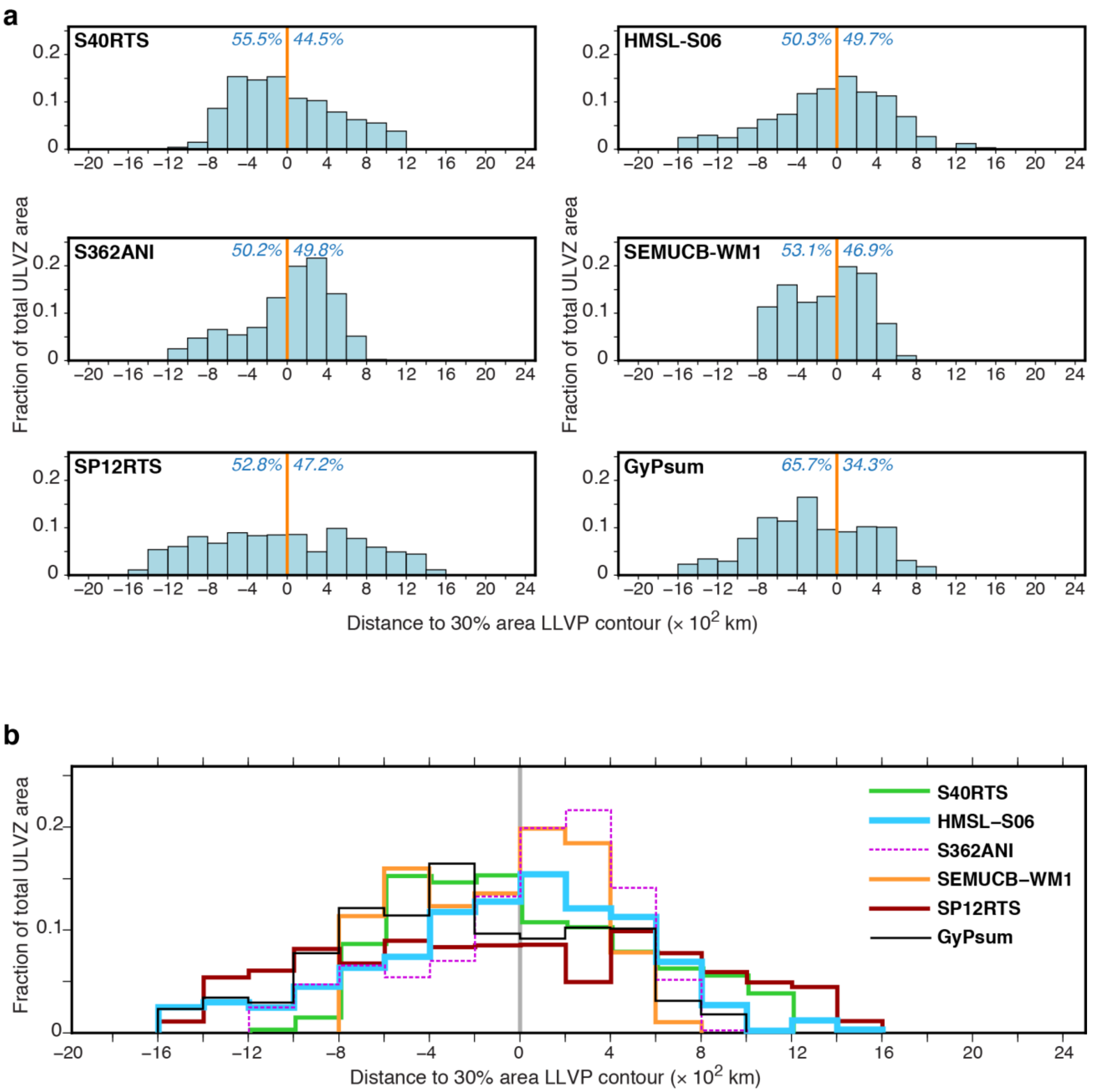

Supplementary Figure 11 | Distance of ULVZs from the closest LLVP edges at 2800 km depth. **a**, similar to Figure 5c in the main text. These panels are differed by the seismic tomography model used to define LLVP edges. **b**, same as **a**, but with all 6 models plotted together for better comparison. For all models, we select 30% CMB area with the lowest seismic velocity, and define this area as the locations of LLVPs.

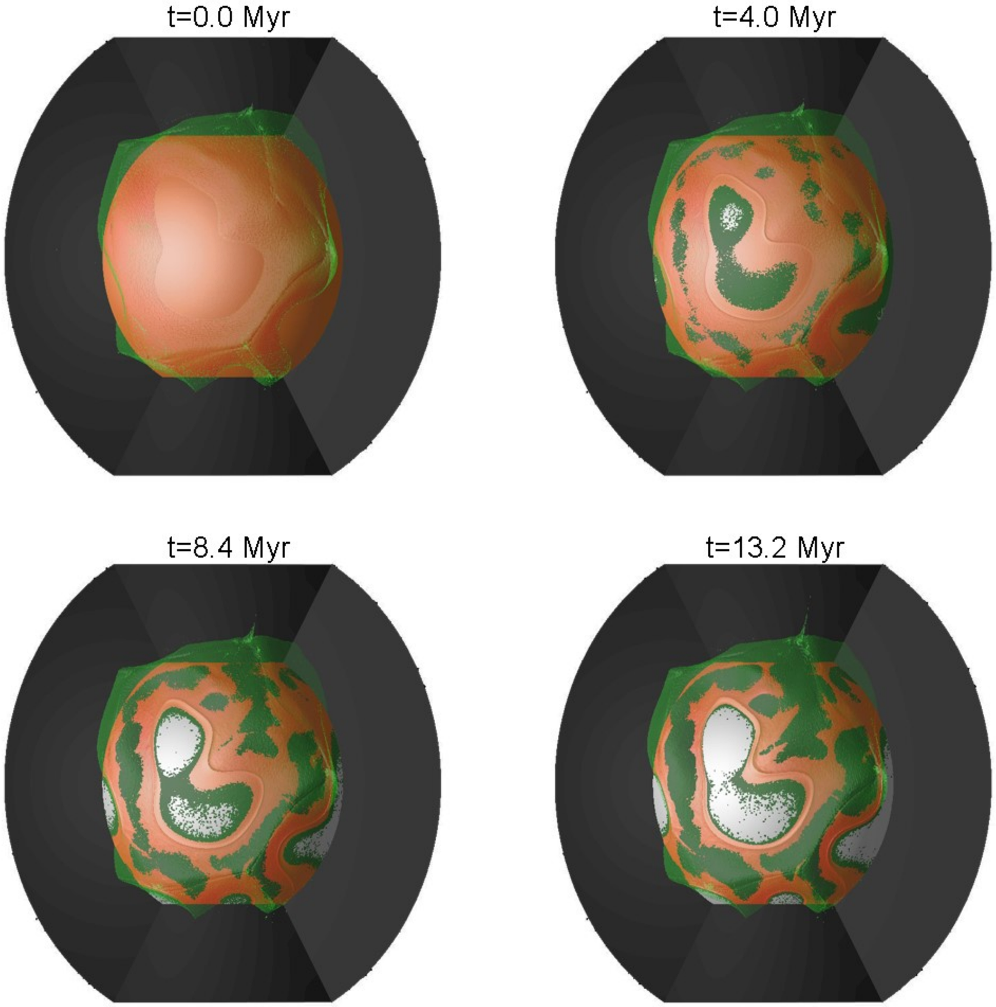

Supplementary Figure 12 | Snapshot of compositional field (viewing from top to bottom) showing the initial migration of a global layer of ultra-dense material (orange) at t=0.0 Myr to the edges of thermochemical piles (green) after ~13 Myrs. The dark gray color shows the computational domain, and the light gray color shows the core. The time above each panel shows the geological time for the snapshot.

Supplementary References

1. Dobson, D. P. & Brodholt, J. P. Subducted banded iron formations as a source of ultralow-velocity zones at the core-mantle boundary. *Nature* **434**, 371-374 (2005).
2. Liu, J., Li, J., Hrubiak, R. & Smith, J. S. Origins of ultralow velocity zones through slab-derived metallic melt. *Proc. Natl. Acad. Sci. U.S.A.* **113**, 5547-5551 (2016).
3. Wicks, J. K., Jackson, J. M. & Sturhahn, W. Very low sound velocities in iron-rich (Mg,Fe)O: Implications for the core-mantle boundary region. *Geophys. Res. Lett.* **37**, L15304 (2010).
4. Boehler, R. Melting of the Fe-FeO and the Fe-FeS systems at high pressure: Constraints on core temperatures. *Earth Planet. Sci. Lett.* **111**, 217-227 (1992).
5. Nomura, R. *et al.* Spin crossover and iron-rich silicate melt in the Earth's deep mantle. *Nature* **473**, 199-202 (2011).
6. Zerr, A. & Boehler, R. Constraints on the melting temperature of the lower mantle from high-pressure experiments on MgO and magnesioustite. *Nature* **371**, 506-508 (1994).
7. Deschamps, F. & Tackley, P. J. Searching for models of thermo-chemical convection that explain probabilistic tomography. *Phys. Earth Planet. Int.* **171**, 357-373 (2008).
8. Deschamps, F. & Tackley, P. J. Searching for models of thermo-chemical convection that explain probabilistic tomography. II--Influence of physical and compositional parameters. *Phys. Earth Planet. Int.* **176**, 1-18 (2009).
9. Zhao, C., Garnero, E. J., Li, M., McNamara, A. & Yu, S. Intermittent and lateral varying ULVZ structure at the northeastern margin of the Pacific LLSVP. *J. Geophys. Res.* **122**, 1198-1220 (2017).

10 Gassner, A., Thomas, C., Krüger, F. & Weber, M. Probing the core–mantle boundary beneath Europe and Western Eurasia: A detailed study using PcP. *Phys. Earth Planet. Int.* **246**, 9-24 (2015).

11 Idehara, K. Structural heterogeneity of an ultra-low-velocity zone beneath the Philippine Islands: Implications for core-mantle chemical interactions induced by massive partial melting at the bottom of the mantle. *Phys. Earth Planet. Int.* **184**, 80-90 (2011).

12 Rost, S. & Thomas, C. High resolution CMB imaging from migration of short-period core reflected phases. *Phys. Earth Planet. Int.* **183**, 143-150 (2010).

13 Hutko, A. R., Lay, T. & Revenaugh, J. Localized double-array stacking analysis of PcP: D" and ULVZ structure beneath the Cocos plate, Mexico, central Pacific, and north Pacific. *Phys. Earth Planet. Int.* **173**, 60-74 (2009).

14 He, Y. & Wen, L. Structural features and shear-velocity structure of the "Pacific Anomaly". *J. Geophys. Res.* **114**, B02309 (2009).

15 Idehara, K., Yamada, A. & Zhao, D. Seismological constraints on the ultralow velocity zones in the lowermost mantle from core-reflected waves. *Phys. Earth Planet. Int.* **165**, 25-46 (2007).

16 Rost, S., Garnero, E. J. & Williams, Q. Fine-scale ultralow-velocity zone structure from high-frequency seismic array data. *J. Geophys. Res.* **111**, B09310 (2006).

17 Avants, M., Lay, T. & Garnero, E. J. A new probe of ULVZ S-wave velocity structure: Array stacking of ScS waveforms. *Geophys. Res. Lett.* **33**, L07314 (2006).

18 Rost, S., Garnero, E. J., Williams, Q. & Manga, M. Seismological constraints on a possible plume root at the core-mantle boundary. *Nature* **435**, 666-669 (2005).

19 Ross, A. R., Thybo, H. & Solidilov, L. N. Reflection seismic profiles of the core-mantle boundary. *J. Geophys. Res.* **109**, B08303 (2004).

20 Koper, K. D. & Pyle, M. L. Observations of PKiKP/PcP amplitude ratios and implications for Earth structure at the boundaries of the liquid core. *J. Geophys. Res.* **109**, B03301 (2004).

21 Rost, S. & Revenaugh, J. Small-scale ultralow-velocity zone structure imaged by ScP. *J. Geophys. Res.* **108**, 2056 (2003).

22 Rost, S. & Revenaugh, J. Seismic Detection of Rigid Zones at the Top of the Core. *Science* **294**, 1911-1914 (2001).

23 Ni, S. & Helmberger, D. V. Horizontal transition from fast to slow structures at the core–mantle boundary; South Atlantic. *Earth Planet. Sci. Lett.* **187**, 301-310 (2001).

24 Havens, E. & Revenaugh, J. A broadband seismic study of the lowermost mantle beneath Mexico: Constraints on ultralow velocity zone elasticity and density. *J. Geophys. Res.* **106**, 30809-30820 (2001).

25 Reasoner, C. & Revenaugh, J. ScP constraints on ultralow-velocity zone density and gradient thickness beneath the Pacific. *J. Geophys. Res.* **105**, 28173-28182 (2000).

26 Garnero, E. J. & Vidale, J. E. ScP; A probe of ultralow velocity zones at the base of the mantle. *Geophys. Res. Lett.* **26**, 377-380 (1999).

27 Kohler, M. D., Vidale, J. E. & Davis, P. M. Complex scattering within D″ observed on the very dense Los Angeles Region Seismic Experiment Passive Array. *Geophys. Res. Lett.* **24**, 1855-1858 (1997).

28 Revenaugh, J. & Meyer, R. Seismic Evidence of Partial Melt Within a Possibly Ubiquitous Low-Velocity Layer at the Base of the Mantle. *Science* **277**, 670-673 (1997).

29 Mori, J. & Helmberger, D. V. Localized boundary layer below the mid-Pacific velocity anomaly identified from a PcP precursor. *J. Geophys. Res.* **100**, 20359-20365 (1995).
